# Supplementary material for: AP-1 and TGFß cooperativity drives non-canonical Hedgehog signaling in resistant basal cell carcinoma
Source: Nat Commun. 2020 Oct 8;11:5079. doi: 10.1038/s41467-020-18762-5 (PMC7546632; doi:10.1038/s41467-020-18762-5)
Supplement: Supplementary file 1 — Supplementary Information [file 41467_2020_18762_MOESM1_ESM.pdf]

Supplementary Information

AP-1 and TGFß cooperativity drives non-canonical Hedgehog signaling in resistant basal cell carcinoma

Catherine D. Yao[1], Daniel Haensel[1], Sadhana Gaddam[1], Tiffany Patel[1], Scott X. Atwood[1,2], Kavita Y. Sarin[1], Ramon J. Whitson[1,3], Siegen McKellar[1,4], Gautam Shankar[1,5], Sumaira Aasi[1], Kerri Rieger[1], and Anthony E. Oro[1,6]

[1]Program in Epithelial Biology, Stanford University School of Medicine, Stanford, CA 94305. Current Address: [2]Department of Developmental and Cell Biology, University of California, Irvine; [3]Genomics Institute of the Novartis Research Foundation, La Jolla, CA; [4]University of Washington School of Medicine, Seattle, WA 98195; [5]Johns Hopkins School of Medicine Baltimore, MD 21287

Supplementary Figures

Supplementary Figure 1. Resistant nMRTF BCC transcriptomic and chromatin accessibility profiles resemble those of hair follicle matrix transit-amplifying cells.

Supplementary Figure 2. LYPD3, TACSTD2, and LY6D mark the resistant nMRTF subpopulation in naïve patient BCCs.

Supplementary Figure 3. Coincident AP-1 and TGFß signaling are required for BCC resistance.

Supplementary Figure 4. AP-1 is sufficient to drive nMRTF and non-canonical Hh signaling through transcription of RhoGEFs.

Supplementary Figure 5. AP-1 activity establishes the chromatin accessibility and Smad3 DNA binding profile of resistant BCC.

Supplementary Tables

Supplementary Table 1: Published sources of ATAC-seq datasets used in Fig. 1c, Supplementary Fig. 1b-c.

Supplementary Table 2: List of mouse primer sequences used for qRT-PCR.

Supplementary Table 3: List of product numbers for anti-mouse siRNA oligonucleotides synthesized by the Sigma MISSION predesigned siRNA system.

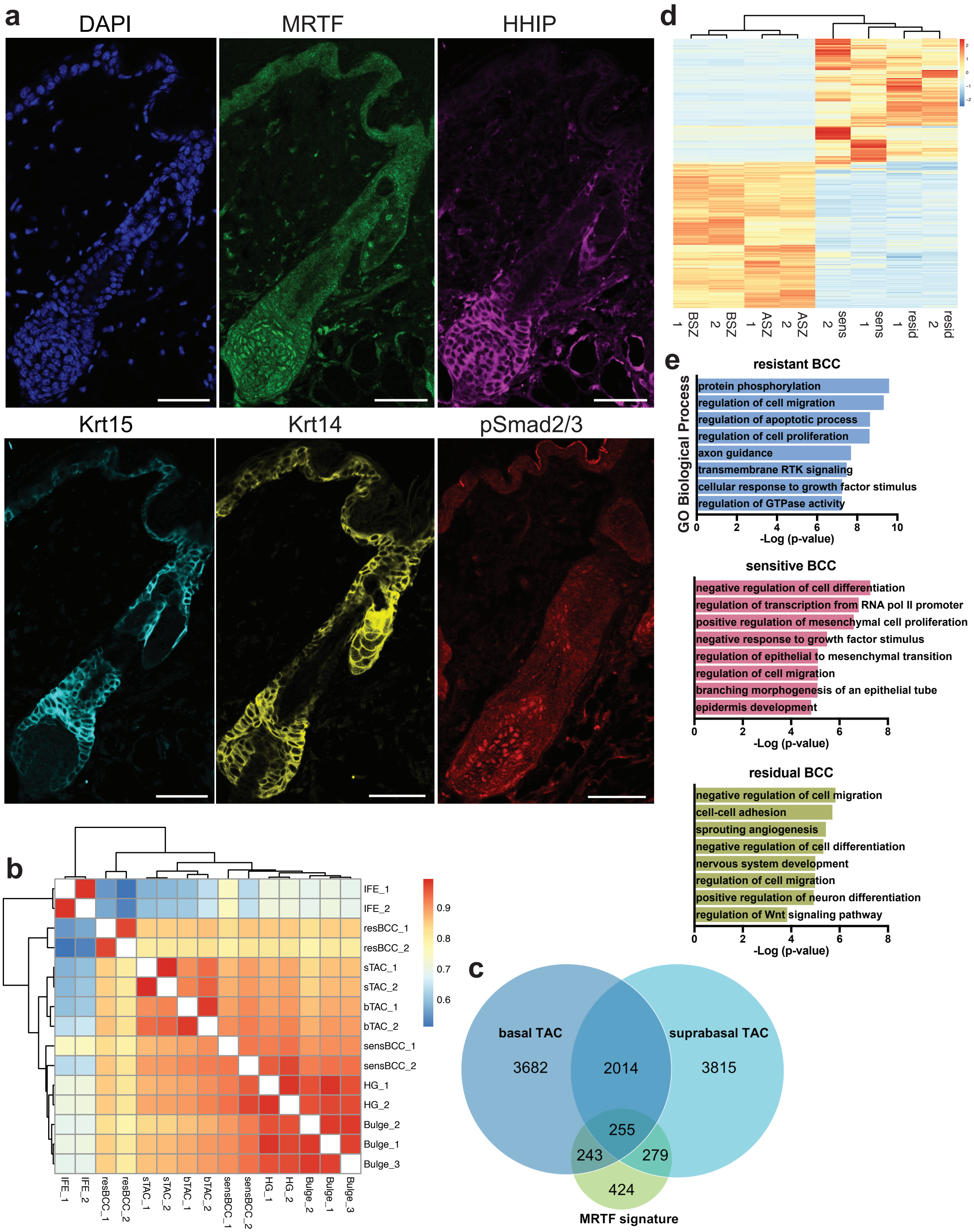
a
DAPI
MRTF
HHIP
Krt15
Krt14
pSmad2/3
b
IFE_1
IFE_2
resBCC_1
resBCC_2
sTAC_1
sTAC_2
bTAC_1
bTAC_2
sensBCC_1
sensBCC_2
HG_1
HG_2
Bulge_2
Bulge_1
Bulge_3
0.9
0.8
0.7
0.6
c
basal TAC
suprabasal TAC
MRTF signature
3682
2014
3815
255
243
279
424
d
BSZ 1
BSZ 2
ASZ 1
ASZ 2
sens 2
sens 1
resid 1
resid 2
e
GO Biological Process
resistant BCC
protein phosphorylation
regulation of cell migration
regulation of apoptotic process
regulation of cell proliferation
axon guidance
transmembrane RTK signaling
cellular response to growth factor stimulus
regulation of GTPase activity
-Log (p-value)
sensitive BCC
negative regulation of cell differentiation
regulation of transcription from RNA pol II promoter
positive regulation of mesenchymal cell proliferation
negative response to growth factor stimulus
regulation of epithelial to mesenchymal transition
regulation of cell migration
branching morphogenesis of an epithelial tube
epidermis development
-Log (p-value)
residual BCC
negative regulation of cell migration
cell-cell adhesion
sprouting angiogenesis
negative regulation of cell differentiation
nervous system development
regulation of cell migration
positive regulation of neuron differentiation
regulation of Wnt signaling pathway
-Log (p-value)

Supplementary Figure 1. Resistant nMRTF BCC transcriptomic and chromatin accessibility profiles resemble those of hair follicle matrix transit-amplifying cells.

a. Larger sized image versions of Figure 1a, showing expression levels of DAPI, MRTF, HHIP, Krt15, Krt14, and pSmad2/3. Images are representative of n > 50 hair follicles examined.
b. Correlation plot of ATAC-seq profiles depicted in Figure 1c, including ASZ001 murine resistant BCC cell line (resBCC), sensitive murine BCC (sensBCC), basal transit-amplifying cells of hair follicle (bTAC), bulge hair follicle stem cells (Bulge), hair germ (HG), interfollicular epidermal stem cells (IFE), and suprabasal transit-amplifying cells of hair follicle (sTAC). See Supplementary Table 1 for data sources.
c. Venn diagram showing overlap of target genes on the MRTF signature list (Supplementary Table 4), and genes associated with basal or suprabasal TAC-specific ATAC signals (source in Supplementary Table 1).
d. Heatmap showing clustering of differential ATAC-seq peaks from resistant BCC cell lines (ASZ and BSZ), sensitive BCCs from *K14-creER;Ptch1*$^{fl/fl}$*;Tp53*$^{f/f}$ mice (sens), and residual BCC after 14 days of vismodegib treatment (resid). Thresholds set at fold change > 3 or < -3 and FDR < 0.01. Color bar shows relative ATAC-seq signal (z-score of normalized read counts).
e. Enriched gene ontology (GO) Biological Process terms for each population in (d). p-values calculated by Fisher exact test.

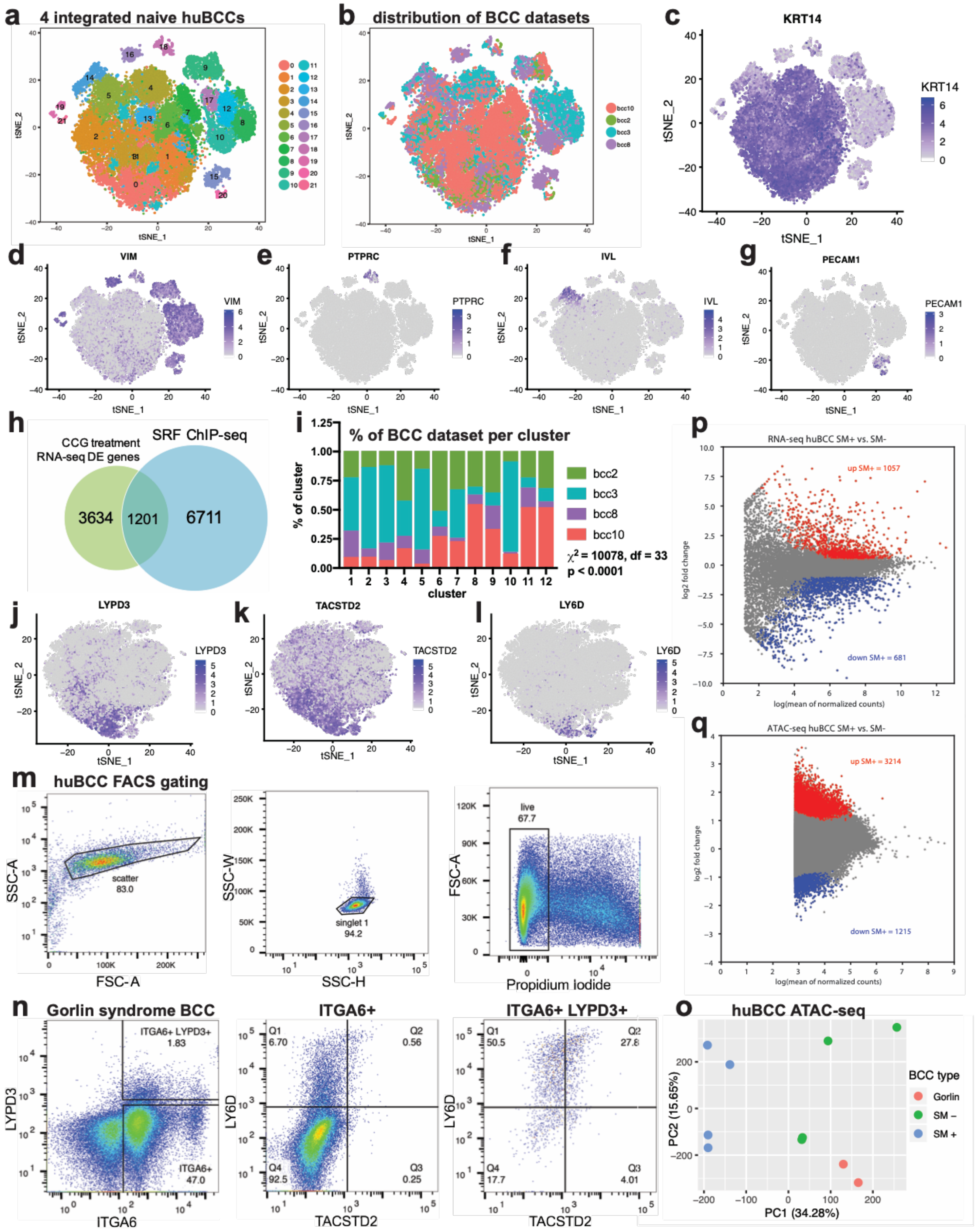

a
4 integrated naive huBCCs
b
distribution of BCC datasets
bcc10
bcc2
bcc3
bcc8
c
KRT14
d
VIM
e
PTPRC
f
IVL
g
PECAM1
h
CCG treatment RNA-seq DE genes
SRF ChIP-seq
3634
1201
6711
i
% of BCC dataset per cluster
% of cluster
cluster
bcc2
bcc3
bcc8
bcc10
χ² = 10078, df = 33
p < 0.0001
j
LYPD3
k
TACSTD2
l
LY6D
m
huBCC FACS gating
scatter
83.0
singlet 1
94.2
live
67.7
SSC-A
FSC-A
SSC-W
SSC-H
FSC-A
Propidium Iodide
n
Gorlin syndrome BCC
ITGA6+ LYPD3+
1.83
ITGA6+
47.0
LYPD3
ITGA6
ITGA6+
Q1
6.70
Q2
0.56
Q4
92.5
Q3
0.25
LY6D
TACSTD2
ITGA6+ LYPD3+
Q1
50.5
Q2
27.8
Q4
17.7
Q3
4.01
LY6D
TACSTD2
o
huBCC ATAC-seq
BCC type
Gorlin
SM –
SM +
PC1 (34.28%)
PC2 (15.65%)
p
RNA-seq huBCC SM+ vs. SM-
up SM+ = 1057
down SM+ = 681
log2 fold change
log(mean of normalized counts)
q
ATAC-seq huBCC SM+ vs. SM-
up SM+ = 3214
down SM+ = 1215
log2 fold change
log(mean of normalized counts)

Supplementary Figure 2. LYPD3, TACSTD2, and LY6D mark the resistant nMRTF subpopulation in naïve patient BCCs.

a. tSNE plot of unbiased clustering for combined scRNA-seq datasets from 4 patient BCC tumors. Clustering conducted post multi-CCA alignment.
b. Distribution of 4 BCC datasets across clusters from (a).
c. Expression distribution of Krt14 marking epithelial cells, vimentin marking fibroblasts (d), PTPRC marking immune cells (e), involucrin marking differentiated keratinocytes (f), and PECAM-1 marking endothelial cells (g) among BCC scRNA-seq clusters.
h. Overlapped gene lists from previous RNA-seq (differentially expressed genes with treatment of MRTF inhibitor CCG-1423) and SRF ChIP-seq (peaks mapped to nearest gene by GREAT) conducted in resistant ASZ001 cells, used to define MRTF-active signature. See also Supplementary Table 4.
i. % distribution of four original BCC datasets in each ranked cluster, post Krt-14 expression filtering. Chi-squared test statistic with p-value shown.
j. Expression distribution of LYPD3, TACSTD2 (k), and LY6D(l) across scRNA-seq clusters of patient BCC tumors filtered for positive Krt14 expression.
m. Representative FACS plots showing initial gating for sorting of naïve human BCC.
n. Representative FACS plots showing expression of ITGA6, LYPD3, TACSTD2, and LY6D in Gorlin syndrome patient BCC.
o. PCA plot of ATAC-seq datasets from ITGA6+ Gorlin patient BCC cells (Gorlin), naïve patient BCC ITGA6+LYPD3+TACSTD2+LY6D+/- (SM+) cells and ITGA6+LYPD3-TACSTD2-LY6D- (SM-) cells.
p. Differential peaks from RNA-seq of human naïve BCC cells sorted SM+ vs. SM-. Significance threshold set at $\log_2$ fold change > 1 or < -1, $p < 0.05$. n = 4 biological replicates from 2 patients.
q. Differential peaks from ATAC-seq of human naïve BCC cells sorted SM+ vs. SM-. Significance threshold set at $\log_2$ fold change > 1 or < -1, $p < 0.05$. n = 4 biological replicates from 2 patients.

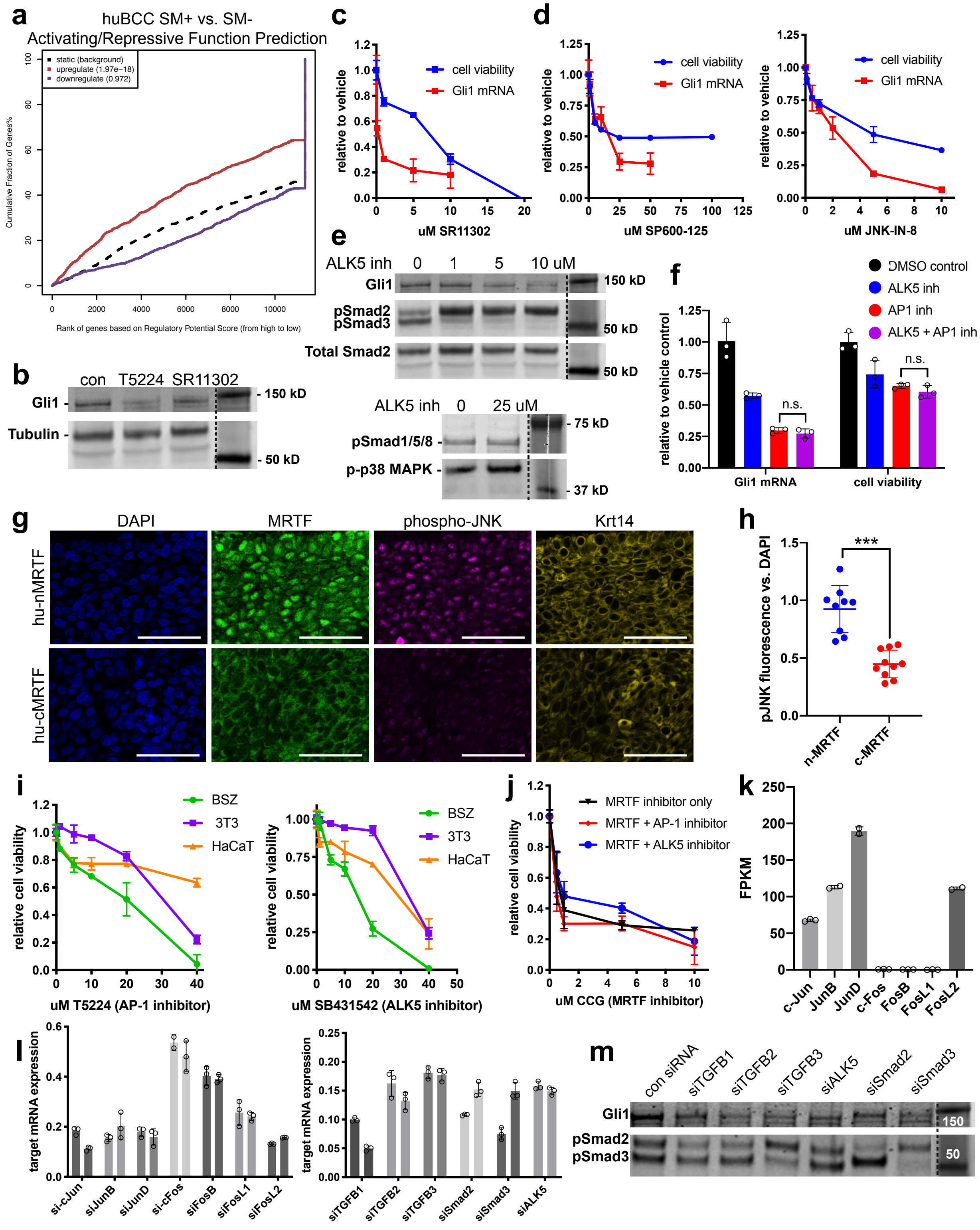

a
huBCC SM+ vs. SM-
Activating/Repressive Function Prediction
static (background)
upregulate (1.97e−18)
downregulate (0.972)
Cumulative Fraction of Genes%
Rank of genes based on Regulatory Potential Score (from high to low)
b
con
T5224
SR11302
Gli1
Tubulin
150 kD
50 kD
c
relative to vehicle
cell viability
Gli1 mRNA
uM SR11302
d
uM SP600-125
uM JNK-IN-8
e
ALK5 inh
0
1
5
10 uM
Gli1
pSmad2
pSmad3
Total Smad2
150 kD
50 kD
ALK5 inh
25 uM
pSmad1/5/8
p-p38 MAPK
75 kD
37 kD
f
DMSO control
ALK5 inh
AP1 inh
ALK5 + AP1 inh
relative to vehicle control
n.s.
Gli1 mRNA
cell viability
g
DAPI
MRTF
phospho-JNK
Krt14
hu-nMRTF
hu-cMRTF
h
pJNK fluorescence vs. DAPI
***
n-MRTF
c-MRTF
i
BSZ
3T3
HaCaT
relative cell viability
uM T5224 (AP-1 inhibitor)
uM SB431542 (ALK5 inhibitor)
j
MRTF inhibitor only
MRTF + AP-1 inhibitor
MRTF + ALK5 inhibitor
uM CCG (MRTF inhibitor)
k
FPKM
c-Jun
JunB
JunD
c-Fos
FosB
FosL1
FosL2
l
target mRNA expression
si-cJun
siJunB
siJunD
si-cFos
siFosB
siFosL1
siFosL2
siTGFB1
siTGFB2
siTGFB3
siSmad2
siSmad3
siALK5
m
con siRNA
siTGFB1
siTGFB2
siTGFB3
siALK5
siSmad2
siSmad3
Gli1
pSmad2
pSmad3
150
50

Supplementary Figure 3. Coincident AP-1 and TGFß signaling are required for BCC resistance.

a. Activating/Repressive Function prediction graph from BETA integration of upregulated ATAC-seq peaks and RNA-seq differential expression analysis in sorted patient BCC SM+ vs. SM- cells.
b. Western blot of ASZ001 cells after 24-hour treatment of AP-1 inhibitors 20 μM T5224 or 5 μM SR11302.
c. *Gli1* qRT-PCR and MTS cell viability assay of ASZ001 cells treated with AP-1 inhibitor SR11302.
d. *Gli1* qRT-PCR and MTS cell viability assays of ASZ001 cells treated with JNK inhibitors SP600-125 or JNK-IN-8.
e. Western blot of ASZ001 cells after 24-hour treatment of 1, 5, 10, or 25 μM of ALK5 inhibitor SB431542.
f. Gli1 qRT-PCR and MTS cell viability assays of ASZ001 cells treated with 10 μM of ALK5 inhibitor SB431542, AP-1 inhibitor T5224, or both.
g. Representative IF images of phospho-JNK in human BCCs with nuclear or cytoplasmic MRTF.
h. Quantification of fluorescence intensity of phospho-JNK vs. DAPI in (g). Each point represents mean pixel intensity, normalized to mean DAPI intensity, averaged over at least three 100x100 micron microscopy fields. *** = $p < 0.0001$.
i. MTS cell viability assays of BSZ, 3T3, or HaCaT cells treated with T5224 or SB431542 for 72 hours.
j. MTS cell viability assay of ASZ001 cells treated with increasing doses of CCG only for 72 hours, or CCG + 10 μM SB431542 or 10 μM T5224.
k. FPKM values of AP-1 subunit mRNA expression in ASZ001 cells.
l. Validation of siRNAs in ASZ001 cells with target gene qRT-PCR. Each pair of matching-colored bars represents two distinct siRNA oligos per target gene. Open circles on all bar graphs represent biological replicates. All error bars represent mean +/- SD. P-values calculated by unpaired, two-tailed Student's t-test.
m. Western blot of ASZ001 cells transfected with TGFB family siRNAs for 48 hours. All western blot images are representative of at least three independent experiments, with far-right columns showing molecular weight markers.

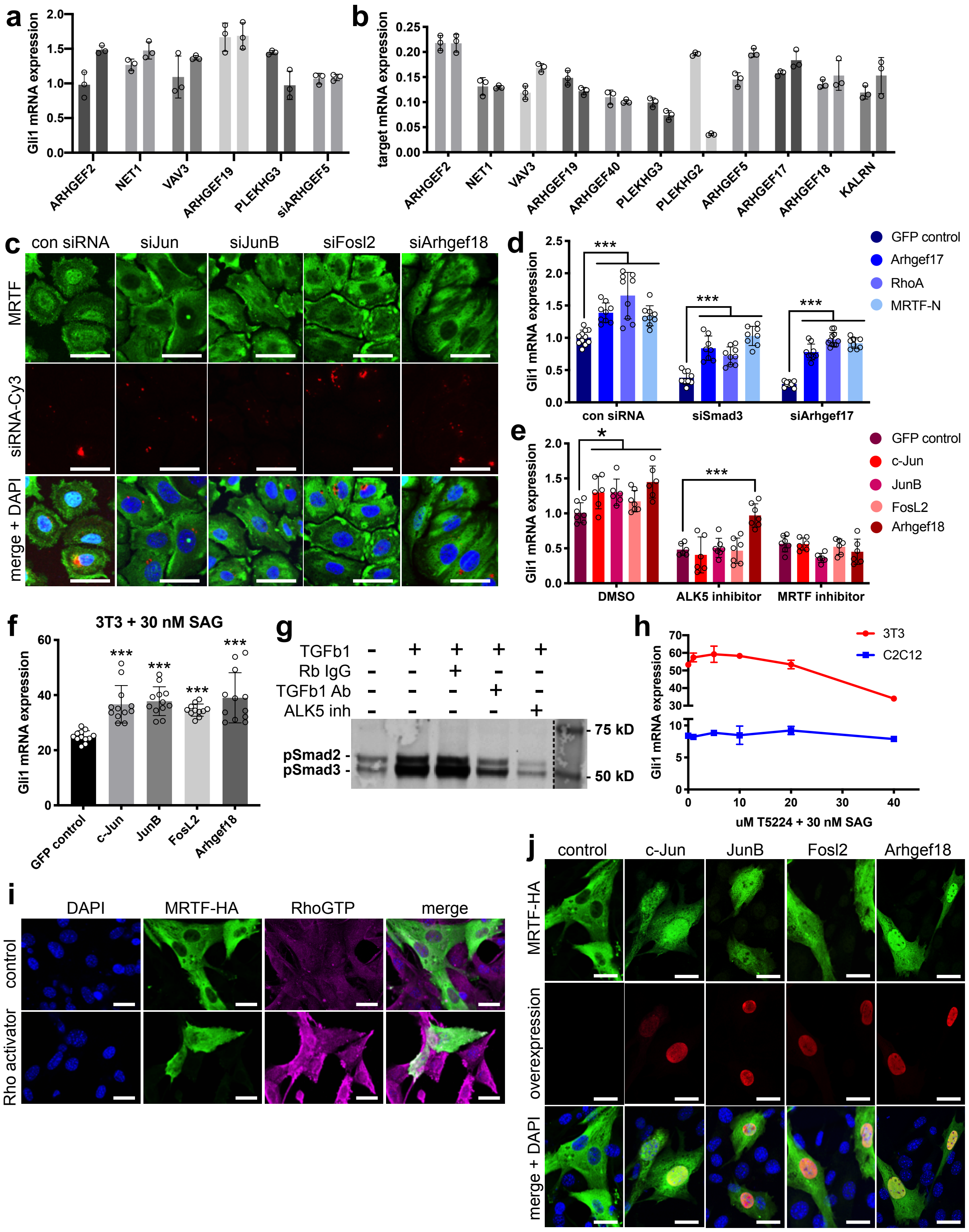

a
Gli1 mRNA expression
ARHGEF2
NET1
VAV3
ARHGEF19
PLEKHG3
siARHGEF5
b
target mRNA expression
ARHGEF2
NET1
VAV3
ARHGEF19
ARHGEF40
PLEKHG3
PLEKHG2
ARHGEF5
ARHGEF17
ARHGEF18
KALRN
c
con siRNA
siJun
siJunB
siFosl2
siArhgef18
MRTF
siRNA-Cy3
merge + DAPI
d
Gli1 mRNA expression
con siRNA
siSmad3
siArhgef17
GFP control
Arhgef17
RhoA
MRTF-N
e
Gli1 mRNA expression
DMSO
ALK5 inhibitor
MRTF inhibitor
GFP control
c-Jun
JunB
FosL2
Arhgef18
f
3T3 + 30 nM SAG
Gli1 mRNA expression
GFP control
c-Jun
JunB
FosL2
Arhgef18
g
TGFb1
Rb IgG
TGFb1 Ab
ALK5 inh
pSmad2
pSmad3
75 kD
50 kD
h
3T3
C2C12
Gli1 mRNA expression
uM T5224 + 30 nM SAG
i
DAPI
MRTF-HA
RhoGTP
merge
control
Rho activator
j
control
c-Jun
JunB
Fosl2
Arhgef18
MRTF-HA
overexpression
merge + DAPI

Supplementary Figure 4. AP-1 is sufficient to drive nMRTF and non-canonical Hh signaling through transcription of RhoGEFs.

a. *Gli1* qRT-PCR of ASZ001 cells transfected with siRNAs targeted selected GEFs.
b. Validation of GEF siRNAs in ASZ001 cells with target gene qRT-PCR. Each pair of matching-colored bars represents two distinct siRNA oligos per target gene.
c. IF of MRTF expression in ASZ001 cells transfected with Cy-3 conjugated siRNAs against AP-1 family members and Arhgef18.
d. Epistatic studies measured by *Gli1* qRT-PCR of ASZ001 cells transfected with siRNAs and overexpression constructs. *** = $p < 0.0001$.
e. Epistatic studies measured by *Gli1* qRT-PCR of ASZ001 cells transfected with AP-1 subunit and Arhgef18 overexpression constructs and treated with inhibitors. * = $p < 0.05$, *** = $p < 0.0001$.
f. *Gli1* qRT-PCR of NIH-3T3 cells transiently transfected with AP-1 subunit and Arhgef18 overexpression constructs. *** = $p < 0.0001$. Open circles on all bar graphs represent biological replicates. All error bars represent mean +/- SD. P-values calculated by unpaired, two-tailed Student's t-test.
g. Western blot of phosphorylated Smad2 and Smad3 protein levels in 3T3 cells stimulated with 1 ng/ml of TGFB1 protein and/or control IgG, anti-TGFB1 antibody, or 10 μM ALK5 inhibitor SB431542. Image representative of at least three independent experiments. Molecular weight markers shown on far right column.
h. *Gli1* qRT-PCR of 3T3 and C2C12 cells treated with 30 nM SAG and increasing doses of AP-1 inhibitor T5224.
i. IF of MRTF localization and RhoGTP levels in 3T3s treated with 1 ug/ml of Rho Activator II for 4 hours.
j. IF of MRTF localization in 3T3s transfected with HA-tagged MRTF and AP-1 subunit or Arhgef18 overexpression constructs. All IF images are representative of n > 50 cells.

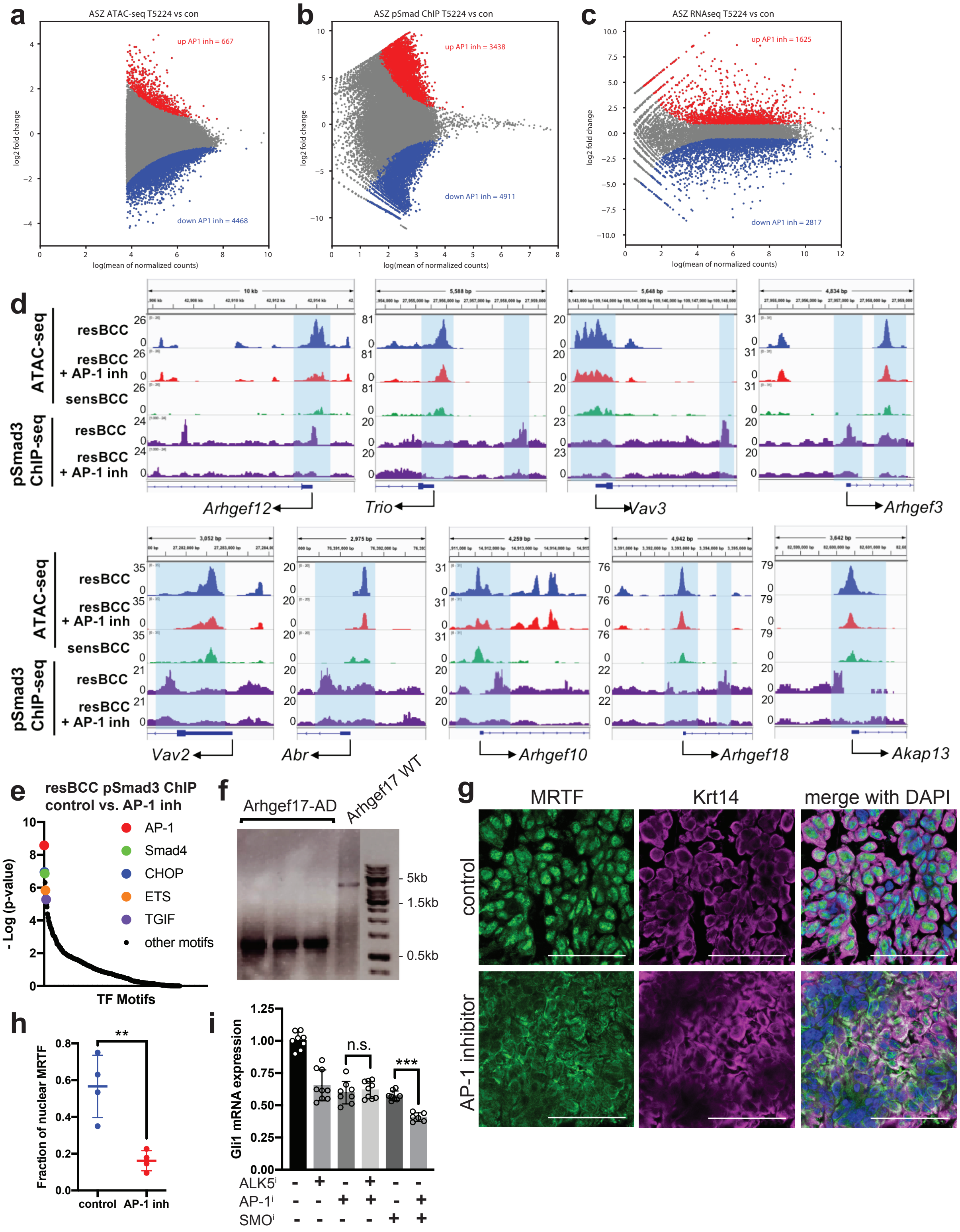
a
ASZ ATAC-seq T5224 vs con
up AP1 inh = 667
down AP1 inh = 4468
log2 fold change
log(mean of normalized counts)
b
ASZ pSmad ChIP T5224 vs con
up AP1 inh = 3438
down AP1 inh = 4911
log2 fold change
log(mean of normalized counts)
c
ASZ RNAseq T5224 vs con
up AP1 inh = 1625
down AP1 inh = 2817
log2 fold change
log(mean of normalized counts)
d
ATAC-seq
resBCC
resBCC + AP-1 inh
sensBCC
pSmad3 ChIP-seq
resBCC
resBCC + AP-1 inh
Arhgef12
Trio
Vav3
Arhgef3
Vav2
Abr
Arhgef10
Arhgef18
Akap13
e
resBCC pSmad3 ChIP control vs. AP-1 inh
- Log (p-value)
TF Motifs
AP-1
Smad4
CHOP
ETS
TGIF
other motifs
f
Arhgef17-AD
Arhgef17 WT
5kb
1.5kb
0.5kb
g
MRTF
Krt14
merge with DAPI
control
AP-1 inhibitor
h
Fraction of nuclear MRTF
**
control
AP-1 inh
i
Gli1 mRNA expression
n.s.
***
ALK5ⁱ
AP-1ⁱ
SMOⁱ

Supplementary Figure 5. AP-1 activity establishes the chromatin accessibility and Smad3 DNA binding profile of resistant BCC.

a. Differential peaks from ATAC-seq of resistant ASZ cells treated with 20 μM AP-1 inhibitor T-5224 for 24 hours. Significance threshold for (a)-(c) set at log_2 fold change > 1 or < -1, $p < 0.05$.
b. Differential peaks from pSmad3 ChIP-seq of ASZ cells treated with 20 μM AP-1 inhibitor T-5224 for 24 hours.
c. Differential gene expression from RNAseq of ASZ cells treated with 20 μM AP-1 inhibitor T-5224 for 24 hours.
d. Representative ATAC-seq and pSmad3 ChIP-seq tracks at RhoGEF loci, visualized in IGV.
e. Transcription factor motifs enriched at resistant ASZ001 Smad3 binding peaks, which are downregulated with AP-1 inhibition (log_2 fold change <-1, $p < 0.05$).
f. Image of agarose gel of PCR products confirming deletion from three separate ASZ001 cell lines generated by CRISPR-Cas9 targeting the first intron of *Arhgef17*, named Arhgef17 ATAC-peak deletion (Arhgef17 AD).
g. Representative IF images of MRTF and Krt14 staining in naïve patient BCC explants treated with DMSO control or 10 μM AP-1 inhibitor SR11302 for 24 hours. Scale bar = 50 μm.
h. Quantification of cells in (g) containing nuclear MRTF in naïve patient BCC explants treated with DMSO control or AP-1 inhibitor. Each point represents fraction of MRTF-nuclear cells out of all Krt-14 positive cells from one tumor sample, averaged over at least three 300 x 300 μm microscopy fields. ** = p = 0.0039.
i. qRT-PCR of Gli1 mRNA expression in naïve patient BCC explants after 24 hour treatment with DMSO control, 40 μM ALK5 inhibitor SB431542, 10 μM AP-1 inhibitor SR11302, 1 μM SMO inhibitor vismodegib, or combinations. *** = $p < 0.0001$, calculated using unpaired, two-tailed Student's t-test. Open circles represent biological replicates. All error bars represent mean +/- SD.

**Sample name**	**Source**	**SRA #s**
sens-BCC	Biehs et. al (2018). A cell identity switch allows residual BCC to survive Hedgehog pathway inhibition. *Nature*, *562*(7727), 429–433	SRR7511801<br>SRR7511804
resid-BCC	Biehs et. al (2018). A cell identity switch allows residual BCC to survive Hedgehog pathway inhibition. *Nature*, *562*(7727), 429–433	SRR7511807<br>SRR7511808
basal TAC	Adam, R. et. al (2018). Temporal Layering of Signaling Effectors Drives Chromatin Remodeling during Hair Follicle Stem Cell Lineage Progression. *Cell Stem Cell*, *22*(3), 398-413.e7.	SRR5808767<br>SRR5808768
suprabasal TAC	Adam, R. et. al (2018). Temporal Layering of Signaling Effectors Drives Chromatin Remodeling during Hair Follicle Stem Cell Lineage Progression. *Cell Stem Cell*, *22*(3), 398-413.e7.	SRR5808769<br>SRR5808770
Hair Germ	Adam, R. et. al (2018). Temporal Layering of Signaling Effectors Drives Chromatin Remodeling during Hair Follicle Stem Cell Lineage Progression. *Cell Stem Cell*, *22*(3), 398-413.e7.	SRR5808765<br>SRR5808766
Bulge	Adam, R. et. al (2018). Temporal Layering of Signaling Effectors Drives Chromatin Remodeling during Hair Follicle Stem Cell Lineage Progression. *Cell Stem Cell*, *22*(3), 398-413.e7.	SRR5808763<br>SRR5808764
Bulge	Ge, Y. et. al (2017). Stem Cell Lineage Infidelity Drives Wound Repair and Cancer. *Cell*, *169*(4), 636-650.e14.	SRR5027055<br>SRR5027054
IFE	Ge, Y. et. al (2017). Stem Cell Lineage Infidelity Drives Wound Repair and Cancer. *Cell*, *169*(4), 636-650.e14.	SRR5027052<br>SRR5027053

Supplementary Table 1: Published sources of ATAC-seq datasets used in Fig. 1c, Supplementary Fig. 1b-c.

Target Gene	Forward sequence	Reverse sequence
Arhgef17	GTCTGGACGACGACTCCAC	CCCACGAAACTCTGTCGGG
Arhgef18	ATGAAGCTGACTCCGTGTTTTT	TCAGTGAAGCAATGTAAGGGTC
Arhgef19	GTCGCCCAGTAGCTGTGTG	CCTCTGGAGCAATGGGAAAGA
Arhgef2	TGATGACAGATGTGCTCGTGT	GCTTGTCCAGAGACGTGAAAAT
Arhgef40	GCCCGTAAGAGCACTGGAG	CCCTGAAGGTGAGTCGGAAG
Arhgef5	GTCAGAAGCATCTTACCTGCG	CGAGAGAAGAGCCACTGATGAT
Ect2	AACTTGTGCTTGGCGTCTACT	TTCCTCCGATTTTCCAGGACA
Fos	CGGGTTTCAACGCCGACTA	TTGGCACTAGAGACGGACAGA
FosB	TTTTCCCGGAGACTACGACTC	GTGATTGCGGTGACCGTTG
Fosl1	ATGTACCGAGACTACGGGGAA	CTGCTGCTGTCGATGCTTG
Fosl2	CCAGCAGAAGTTCCGGGTAG	GTAGGGATGTGAGCGTGGATA
Gli1	CCAAGCCAACTTTATGTCAGGG	AGCCCGCTTCTTTGTTAATTTGA
Jun	CCTTCTACGACGATGCCCTC	GGTTCAAGGTCATGCTCTGTTT
JunB	TCACGACGACTCTTACGCAG	CCTTGAGACCCCGATAGGGA
JunD	GAAACGCCCTTCTATGGCGA	CAGCGCGTCTTTCTTCAGC
Kalrn	CCGACTCTTGGACTACCTTATGA	CGACCCTACAGTTGTGCAG
Net1	CGGCGAACGAGAGATGCTC	CTCCTTCAAATCAAGGCTGCTA
Plekhg2	GAATCCAGGGTTCGGTGAAAC	AATCTTCGCTTCTTTGCACGA
Plekhg3	AGGTTGAGACAGATCCTGAGC	GGGTTGTCCACTCCTAGCAG
Serpine1	TTCAGCCCTTGCTTGCCTC	ACACTTTTACTCCGAAGTCGGT
Smad2	ATGTCGTCCATCTTGCCATTC	AACCGTCCTGTTTTCTTTAGCTT
Smad3	CATTCCATTCCCGAGAACACTAA	GCTGTGGTTCATCTGGTGGT
Spata13	GTTAGGCTTCGAGTCAATCAGG	ATGACGTTGGTCCGCATCTG
Tgfb1	CTCCCGTGGCTTCTAGTGC	GCCTTAGTTTGGACAGGATCTG
Tgfb2	CTTCGACGTGACAGACGCT	GCAGGGGCAGTGTAAACTTATT
Tgfb3	CCTGGCCCTGCTGAACTTG	TTGATGTGGCCGAAGTCCAAC
Tgfbr1	TCTGCATTGCACTTATGCTGA	AAAGGGCGATCTAGTGATGGA
Vav3	CCTGCTGCGATACCTTTGGAA	GTGTTCGGGATAGCCGAGATA

Supplementary Table 2: List of mouse primer sequences used for qRT-PCR.

Target Gene	Set 1	Set 2	Set 3
Arhgef17	SASI_Mm02_00295341	SASI_Mm02_00295342	SASI_Mm02_00295343
Arhgef18	SASI_Mm01_00196299	SASI_Mm01_00196300	SASI_Mm02_00339079
Arhgef19	SASI_Mm01_00059936	SASI_Mm01_00059937	SASI_Mm01_00059938
Arhgef2	SASI_Mm01_00087177	SASI_Mm02_00314012	SASI_Mm01_00087179
Arhgef40	SASI_Mm01_00026546	SASI_Mm02_00350899	SASI_Mm01_00026547
Arhgef5	SASI_Mm02_00338623	SASI_Mm02_00338624	SASI_Mm02_00338625
Ect2	SASI_Mm01_00046597	SASI_Mm01_00046599	SASI_Mm01_00046600
Fos	SASI_Mm01_00192758	SASI_Mm01_00192759	SASI_Mm01_00192760
FosB	SASI_Mm01_00145146	SASI_Mm01_00145147	SASI_Mm01_00145148
Fosl1	SASI_Mm01_00062525	SASI_Mm01_00062526	SASI_Mm02_00318095
Fosl2	SASI_Mm01_00201000	SASI_Mm01_00201001	SASI_Mm01_00201002
Jun	SASI_Mm01_00046356	SASI_Mm01_00046357	SASI_Mm01_00046358
JunB	SASI_Mm01_00103237	SASI_Mm01_00103243	SASI_Mm02_00313865
JunD	SASI_Mm02_00318898	SASI_Mm01_00184444	SASI_Mm01_00184443
Kalrn	SASI_Mm02_00355143	SASI_Mm02_00355144	SASI_Mm02_00355145
Net1	SASI_Mm02_00291891	SASI_Mm02_00291892	SASI_Mm02_00291893
Plekhg2	SASI_Mm02_00299961	SASI_Mm02_00299962	SASI_Mm02_00299963
Plekhg3	SASI_Mm01_00164131	SASI_Mm01_00164132	SASI_Mm01_00164133
Smad2	SASI_Mm01_00022386	SASI_Mm01_00022387	SASI_Mm01_00022388
Smad3	SASI_Mm02_00323533	SASI_Mm01_00153031	SASI_Mm01_00153032
Spata13	SASI_Mm01_00233354	SASI_Mm01_00233355	SASI_Mm02_00430211
Tgfb1	SASI_Mm01_00114870	SASI_Mm02_00320969	SASI_Mm01_00114871
Tgfb2	SASI_Mm01_00038392	SASI_Mm01_00038393	SASI_Mm01_00038394
Tgfb3	SASI_Mm02_00315974	SASI_Mm01_00180187	SASI_Mm01_00180188
Tgfbr1	SASI_Mm01_00169048	SASI_Mm01_00169049	SASI_Mm01_00169050
Vav3	SASI_Mm01_00042662	SASI_Mm01_00042663	SASI_Mm01_00042664

Supplementary Table 3: List of product numbers for anti-mouse siRNA oligonucleotides synthesized by the Sigma MISSION predesigned siRNA system.
