# Supplementary material for: AP-1 and TGFß cooperativity drives non-canonical Hedgehog signaling in resistant basal cell carcinoma
Source: Nat Commun. 2020 Oct 8;11:5079. doi: 10.1038/s41467-020-18762-5 (PMC7546632; doi:10.1038/s41467-020-18762-5)
Supplement: Supplementary file 3 — Description of Additional Supplementary Files [file 41467_2020_18762_MOESM3_ESM.pdf]

Description of Additional Supplementary Files

Title: Supplementary Data 1

Description: List of MRTF signature genes generated by intersection of SRF target genes by ChIP-seq and genes downregulated with MRTF inhibition by RNA-seq (log2FC < -1 and p < 0.05) in resistant BCC cells. Used in Fig. 2 and Supplementary Fig. 2.

Title: Supplementary Data 2

Description: Intersection of genes downregulated by RNA-seq in resistant BCC cells after treatment with AP-1 inhibitor and ALK5 inhibitor, used in Fig. 4. Significance cutoffs of log2FC < -1 and adjusted p-value < 0.05.

Title: Supplementary Data 3

Description: Intersection of genes that are downregulated with AP-1 inhibition (log2FC < -1 and p < 0.05) in resistant BCC cells as measured by RNA-seq, ATAC-seq, and pSmad3 ChIP-seq. Used in Figure 6 and Supplementary Figure 5.
