# Supplementary material for: AP-1 and TGFß cooperativity drives non-canonical Hedgehog signaling in resistant basal cell carcinoma
Source: Nat Commun. 2020 Oct 8;11:5079. doi: 10.1038/s41467-020-18762-5 (PMC7546632; doi:10.1038/s41467-020-18762-5)
Supplement: Supplementary file 7 — Reporting Summary [file 41467_2020_18762_MOESM7_ESM.pdf]

nature research

Corresponding author(s): Anthony Oro
Last updated by author(s): Aug 25, 2020

Reporting Summary

Nature Research wishes to improve the reproducibility of the work that we publish. This form provides structure for consistency and transparency in reporting. For further information on Nature Research policies, see our Editorial Policies and the Editorial Policy Checklist.

Statistics

For all statistical analyses, confirm that the following items are present in the figure legend, table legend, main text, or Methods section.

n/a	Confirmed	
☐	☒	The exact sample size (*n*) for each experimental group/condition, given as a discrete number and unit of measurement
☐	☒	A statement on whether measurements were taken from distinct samples or whether the same sample was measured repeatedly
☐	☒	The statistical test(s) used AND whether they are one- or two-sided<br>*Only common tests should be described solely by name; describe more complex techniques in the Methods section.*
☒	☐	A description of all covariates tested
☐	☒	A description of any assumptions or corrections, such as tests of normality and adjustment for multiple comparisons
☐	☒	A full description of the statistical parameters including central tendency (e.g. means) or other basic estimates (e.g. regression coefficient) AND variation (e.g. standard deviation) or associated estimates of uncertainty (e.g. confidence intervals)
☐	☒	For null hypothesis testing, the test statistic (e.g. *F*, *t*, *r*) with confidence intervals, effect sizes, degrees of freedom and *P* value noted<br>*Give P values as exact values whenever suitable.*
☒	☐	For Bayesian analysis, information on the choice of priors and Markov chain Monte Carlo settings
☒	☐	For hierarchical and complex designs, identification of the appropriate level for tests and full reporting of outcomes
☐	☒	Estimates of effect sizes (e.g. Cohen's *d*, Pearson's *r*), indicating how they were calculated

Our web collection on statistics for biologists contains articles on many of the points above.

Software and code

Policy information about availability of computer code

Data collection	Commercial/open source software used for data collection include MxPro v4.1, BD FACSDiva 8.0.1, Leica LAS X v3.7.2, Illumina HiSeq v2.2 and Nextseq System Suite v2.2.0, Softmax Pro 7.1, and LI-COR Image Studio v3.1.
Data analysis	Commercial/open source software used for data analysis include Graphpad Prism 8.0, ImageJ v2.0.0, 10X Genomics Cell Ranger 2.1.1, R v3.4.4, Seurat v2.3.4, TopHat 2.1.1, Homer v4.10, DESeq2, Enrichr, GSEA v3.0, Bowtie 1.1.2, Samtools v0.1.18, MACS2 v2.2.5, GREAT v4.0.4, IGV v2.7, Java Treeview 1.1.6, FlowJo v10.6, BETA v1.0.0, and Image Studio Lite 4.0

For manuscripts utilizing custom algorithms or software that are central to the research but not yet described in published literature, software must be made available to editors and reviewers. We strongly encourage code deposition in a community repository (e.g. GitHub). See the Nature Research guidelines for submitting code & software for further information.

Data

Policy information about availability of data

All manuscripts must include a data availability statement. This statement should provide the following information, where applicable:
- Accession codes, unique identifiers, or web links for publicly available datasets
- A list of figures that have associated raw data
- A description of any restrictions on data availability

Sequencing data generated for this manuscript are available in the Gene Expression Omnibus using GEO accession numbers GSE142469 [https://www.ncbi.nlm.nih.gov/geo/query/acc.cgi?acc=GSE142469] and GSE141526 [https://www.ncbi.nlm.nih.gov/geo/query/acc.cgi?acc=GSE141526]. Previously published sequencing datasets used for analysis for this manuscript are available in the Gene Expression Omnibus using GEO accession numbers GSE100876 [https://www.ncbi.nlm.nih.gov/geo/query/acc.cgi?acc=GSE100876], GSE116966 [https://www.ncbi.nlm.nih.gov/geo/query/acc.cgi?acc=GSE116966], and GSE89928 [https://www.ncbi.nlm.nih.gov/geo/query/acc.cgi?acc=GSE89928]. In addition, we utilized GRch38 and mm9 reference genomes.

Field-specific reporting

Please select the one below that is the best fit for your research. If you are not sure, read the appropriate sections before making your selection.

☒ Life sciences ☐ Behavioural & social sciences ☐ Ecological, evolutionary & environmental sciences

For a reference copy of the document with all sections, see nature.com/documents/nr-reporting-summary-flat.pdf

Life sciences study design

All studies must disclose on these points even when the disclosure is negative.

Sample size	Sample sizes were chosen for at least n = 3 biological replicates. Exact sample sizes are noted in the Methods section. No sample size calculation was performed. Enough replicates were provided to allow for p-value calculations.
Data exclusions	No data were excluded from the analyses.
Replication	Experiments were repeated at least 3 times independently. When possible, alternative methods (different inhibitors, siRNA oligo sequences, etc) were used to confirm specificity of effects. All attempts at replication were successful.
Randomization	Samples were randomly distributed into treatment or control groups and experiments were carried out in parallel.
Blinding	Investigator was not blinded to group allocation because the same investigator conducted data collection as well as analysis.

Reporting for specific materials, systems and methods

We require information from authors about some types of materials, experimental systems and methods used in many studies. Here, indicate whether each material, system or method listed is relevant to your study. If you are not sure if a list item applies to your research, read the appropriate section before selecting a response.

Materials & experimental systems

n/a	Involved in the study	
☐	☒	Antibodies
☐	☒	Eukaryotic cell lines
☒	☐	Palaeontology and archaeology
☐	☒	Animals and other organisms
☐	☒	Human research participants
☒	☐	Clinical data
☒	☐	Dual use research of concern

Methods

n/a	Involved in the study	
☐	☒	ChIP-seq
☐	☒	Flow cytometry
☒	☐	MRI-based neuroimaging

Antibodies

Antibodies used	anti–Krt14 (BioLegend SIG-3476-100), anti-MKL1/MRTF (Novus NBP1-88498), anti-HHIP (Santa Cruz Technologies sc-293265), anti Krt15 (Origene BP5077), anti phospho-Smad2/3 (Abcam ab52903), anti-Ki-67 (Invitrogen MA5-14520), anti-LYPD3 (R&D Systems AF5567), anti-TACSTD2/Trop2 (abcam ab214488), anti-LY6D (Proteintech 17361-1-AP), anti-Arhgef17 (Lifespan Biosciences LS-C385259), anti-phosphoJNK (Invitrogen PA1-9594), and anti-HA tag (abcam ab130275), anti–goat Alexa Fluor 488 (Life Technologies, A-11055), anti–mouse Alexa Fluor 488 (Life Technologies, A-21202), anti–rabbit Alexa Fluor 555 (Life Technologies, A-31572), anti–mouse Alexa Fluor 594 (Life Technologies, A-21203), anti-guinea pig Alexa Fluor 594 (Life Technologies A-11076), and anti–chicken Alexa Fluor 647 (Jackson Immuno Research, 703-606-155), anti-LYPD3 (Sino Biological 11836-R213-P), anti-Trop2/TACSTD2 (R&D systems FAB650-V), anti-LY6D (E48, courtesy of Dr. Ruud Brakenhoff), and anti-CD49f/ItgA6 (Millipore MAB1378), GLI1 (Cell Signaling, 2534), !-tubulin (Developmental Studies Hybridoma Bank, E7), phospho-Smad2/3 (Abcam ab52903), Smad2 (BD Biosciences 610842), phospho-p38 MAPK (Cell Signaling, 4511), phospho-Smad1/5/8 (Cell Signaling, 9511).
Validation	anti–Krt14 (BioLegend SIG-3476-100): https://www.biolegend.com/en-us/products/keratin-14-polyclonal-chicken-antibody-purified-10954. Citation: Papafotiou G, et al. 2016. Nat Commun. 7:11914. Control tested for immunohistochemical staining.<br><br>anti-MKL1/MRTF (Novus NBP1-88498): https://www.novusbio.com/products/mkl1-antibody_nbp1-88498Validated through Novus 'Genetic Strategies' through knockdown. Tested for both western blotting and immunohistochemical staining.<br><br>anti-HHIP (Santa Cruz Technologies sc-293265): https://www.scbt.com/p/hhip-antibody-5d11. Niu C, Chen Z, Kim KT, et al. Metformin alleviates hyperglycemia-induced endothelial impairment by downregulating autophagy via the Hedgehog pathway.

Autophagy. 2019;15(5):843-870. Recommended for western blotting, IP, and immunohistochemical staining.

anti Krt15 (Origene BP5077): https://www.origene.com/catalog/antibodies/primary-antibodies/bp5077/cytokeratin-15-krt15-guinea-pig-polyclonal-antibody. Torkamani N, Rufaut NW, Jones L, Sinclair R. Epidermal Cells Expressing Putative Cell Markers in Nonglabrous Skin Existing in Direct Proximity with the Distal End of the Arrector Pili Muscle. Stem Cells Int. 2016;2016:1286315. Recommended for immunohistochemical staining.

anti phospho-Smad2/3 (Abcam ab52903): https://www.abcam.com/smad3-phospho-s423--s425-antibody-ep823y-ab52903.html. Qu S, Yang L, Liu Z. MicroRNA-194 reduces inflammatory response and human dermal microvascular endothelial cells permeability through suppression of TGF-β/SMAD pathway by inhibiting THBS1 in chronic idiopathic urticaria. J Cell Biochem. 2020;121 (1):111-124. Recommended for western blot, and immunohistochemical staining.

anti-Ki-67 (Invitrogen MA5-14520): https://www.thermofisher.com/antibody/product/Ki-67-Antibody-clone-SP6-Recombinant-Monoclonal/MA5-14520. Invitrogen validation: This Antibody was verified by Cell treatment to ensure that the antibody binds to the antigen stated.

anti-LYPD3 (R&D Systems AF5567): https://www.rndsystems.com/products/mouse-c44a-lypd3-antibody_af5567. Jacobsen, B. and M. Ploug (2008) Curr. Med. Chem. 15:2559. Recommended for western blot, and immunohistochemical staining.

anti-TACSTD2/Trop2 (abcam ab214488): https://www.abcam.com/trop2-antibody-epr20043-ab214488.html. Hou J, Lv A, Deng Q, Zhang G, Hu X, Cui H. TROP2 promotes the proliferation and metastasis of glioblastoma cells by activating the JAK2/STAT3 signaling pathway. Oncol Rep. 2019;41(2):753-764. Recommended for western blot, and immunohistochemical staining.

anti-LY6D (Proteintech 17361-1-AP): https://www.ptglab.com/products/LY6D-Antibody-17361-1-AP.htm#top. Pepe-Mooney BJ, Dill MT, Alemany A, et al. Single-Cell Analysis of the Liver Epithelium Reveals Dynamic Heterogeneity and an Essential Role for YAP in Homeostasis and Regeneration. Cell Stem Cell. 2019;25(1):23-38.e8. Recommended for western blot, and immunohistochemical staining.

anti-Arhgef17 (Lifespan Biosciences LS-C385259): https://www.lsbio.com/antibodies/arhgef17-antibody-tem4-antibody-400-480-aa-internal-elisa-if-immunofluorescence-ihc-ls-c385259/397360. Recommended for ELISA, western blot, and immunohistochemical staining.

anti-phosphoJNK (Invitrogen PA1-9594): https://www.thermofisher.com/antibody/product/Phospho-JNK1-JNK2-Thr183-Tyr185-Antibody-Polyclonal/PA1-9594. Xin JL, Zhang Y, Li Y, Zhang LZ, Lin Y, Zheng LW. Protective effects of Cervus nippon Temminck velvet antler polypeptides against MPP+-induced cytotoxicity in SH-SY5Y neuroblastoma cells. Mol Med Rep. 2017;16(4):5143-5150. Recommended for ELISA, western blot, and immunohistochemical staining.

and anti-HA tag (abcam ab130275): https://www.abcam.com/ha-tag-antibody-16b12-ab130275.html. Antibody no longer available but can still access the data sheet. Recommended for IP, western blot, and immunohistochemical staining.

anti-LYPD3 (Sino Biological 11836-R213-P): https://www.sinobiological.com/antibodies/human-lypd3-11836-r213-p. Wang, C et al.A human monoclonal antibody blocking SARS-CoV-2 infection. Nature Communications.2020. Recommended for flow cytometry.

anti-Trop2/TACSTD2 (R&D systems FAB650-V): https://www.rndsystems.com/products/human-trop-2-alexa-fluor-405-conjugated-antibody-77220_fab650v. Linnenbach, A.J. et al. (1989) Proc. Natl. Acad. Sci. USA 86:27. Recommended for flow cytometry.

anti-LY6D (E48, courtesy of Dr. Ruud Brakenhoff): Brakenhoff RH, Gerretsen M, Knippels EM, et al. The human E48 antigen, highly homologous to the murine Ly-6 antigen ThB, is a GPI-anchored molecule apparently involved in keratinocyte cell-cell adhesion. J Cell Biol. 1995;129(6):1677-1689. doi:10.1083/jcb.129.6.1677

anti-CD49f/ItgA6 (Millipore MAB1378): https://www.emdmillipore.com/US/en/product/Anti-Integrin-6-Antibody-clone-NKI-GoH3,MM_NF-MAB1378. Pageon H, Zucchi H, Dai Z, et al. Biological Effects Induced by Specific Advanced Glycation End Products in the Reconstructed Skin Model of Aging. Biores Open Access. 2015;4(1):54-64. Recommended for flow cytometry, and immunohistochemical staining.

GLI1 (Cell Signaling, 2534): https://www.cellsignal.com/products/primary-antibodies/gli1-v812-antibody/2534. Adams CR, Htwe HH, Marsh T, et al. Transcriptional control of subtype switching ensures adaptation and growth of pancreatic cancer. Elife. 2019;8:e45313. Recommended usage for western blotting.

beta-tubulin (Developmental Studies Hybridoma Bank, E7): https://dshb.biology.uiowa.edu/E7_2. Chu DT, Klymkowsky MW. The appearance of acetylated alpha-tubulin during early development and cellular differentiation in Xenopus. Dev Biol. 1989;136 (1):104-117. Recommended for western blot and immunohistochemical staining.

phospho-Smad2/3 (Abcam ab52903): https://www.abcam.com/smad3-phospho-s423--s425-antibody-ep823y-ab52903.html. Qu S, Yang L, Liu Z. MicroRNA-194 reduces inflammatory response and human dermal microvascular endothelial cells permeability through suppression of TGF-β/SMAD pathway by inhibiting THBS1 in chronic idiopathic urticaria. J Cell Biochem. 2020;121(1):111-124. Recommended for western blot and immunohistochemical staining.

Smad2 (BD Biosciences 610842): https://www.bdbiosciences.com/eu/applications/research/apoptosis/purified-antibodies/purified-mouse-anti-smad23-18smad23/p/610842. Dzwonek J, Preobrazhenska O, Cazzola S, et al. Smad3 is a key nonredundant mediator of transforming growth factor beta signaling in Nme mouse mammary epithelial cells. Mol Cancer Res. 2009;7(8):1342-1353. Recommended for western blot and immunohistochemical staining.

phospho-p38 MAPK (Cell Signaling, 4511): https://www.cellsignal.com/products/primary-antibodies/phospho-p38-mapk-thr180-tyr182-d3f9-xp-rabbit-mab/4511. Rouse, J. et al. (1994) Cell 78, 1027-37. Recommended for western blot, IP, flow cytometry, and immunohistochemical staining.

phospho-Smad1/5/8 (Cell Signaling, 9511): https://www.cellsignal.com/products/primary-antibodies/phospho-smad1-ser463-465-smad5-ser463-465-smad9-ser465-467-antibody/9511. Hogan, B.L. (1996) Genes Dev 10, 1580-94. Recommended for western blot, IP, and immunohistochemical staining.

Eukaryotic cell lines

Policy information about cell lines

Cell line source(s)	ASZ001 (Dr. Ervin Epstein), BSZ2 (Dr. Ervin Epstein), NIH-3T3 (ATCC), C2C12 (ATCC), HaCaT (ATCC).
Authentication	NIH-3T3, C2C12, and HaCaT cell lines were authenticated by ATCC. ASZ001 and BSZ2 cells were authenticated previously by the Epstein group (So et al, Experimental Dermatology 2006). No additional authentication was performed.
Mycoplasma contamination	All cell lines tested negative for mycoplasma contamination.
Commonly misidentified lines (See ICLAC register)	No commonly misidentified cell lines were used in the study.

Animals and other organisms

Policy information about studies involving animals; ARRIVE guidelines recommended for reporting animal research

Laboratory animals	Female C57BL/6J mice were used at p26.
Wild animals	No wild animals were used in the study.
Field-collected samples	No field collected samples were used in the study.
Ethics oversight	The protocol was approved by the Institutional Animal Care and Use Committee (IACUC) at Stanford University.

Note that full information on the approval of the study protocol must also be provided in the manuscript.

Human research participants

Policy information about studies involving human research participants

Population characteristics	Patient population included patients with diagnosed basal cell carcinoma with no prior treatment. Samples were de-identified<br>and there was no selection for age, gender, or other characteristics.
Recruitment	Patients with scheduled Mohs surgery at Stanford Dermatological Surgery clinic were voluntarily recruited with informed consent. No perceived selection bias. Sample availability was dependent on patients willingness to participate in the study.
Ethics oversight	Study protocol was approved by Stanford University IRB.

Note that full information on the approval of the study protocol must also be provided in the manuscript.

ChIP-seq

Data deposition

- [x] Confirm that both raw and final processed data have been deposited in a public database such as GEO.
- [x] Confirm that you have deposited or provided access to graph files (e.g. BED files) for the called peaks.

Data access links<br>*May remain private before publication.*	https://www.ncbi.nlm.nih.gov/geo/query/acc.cgi?acc=GSE142469
Files in database submission	TGFB_pSmad3-ChIPseq_rep1, TGFB_pSmad3-ChIPseq_rep2, T5224_pSmad3-ChIPseq_rep1, T5224_pSmad3-ChIPseq_rep2, input_pSmad3-ChIPseq_rep1, input_pSmad3-ChIPseq_rep2
Genome browser session (e.g. UCSC)	No longer applicable.

April 2020

Methodology

Replicates	Two biological replicates were used per experimental condition.
Sequencing depth	Single-ended 75 bp reads were sequenced to a depth of approximately 20-30 million uniquely mapped reads per sample.
Antibodies	anti-phosphoSmad2/3 (abcam ab52903)
Peak calling parameters	Sequencing reads were mapped to mm9 using Bowtie1.1.2 with parameters –best,–strata and –m 1. Narrow peaks were called using MACS2 with input controls and an FDR threshold 0.05.
Data quality	Read quality was verified using FastQC. Background removal was carried out via submitting replicates to irreproducible discovery rate (IDR) filtering. Number of peaks called ranged from 6179 to 10,447 per sample.
Software	DNA was sequenced using the Illumina Nextseq platform. Reads were mapped using Bowtie 1.1.2 and peaks were called using MACS2. Differential peaks were determined using DESeq2.

Flow Cytometry

Plots

Confirm that:

- [x] The axis labels state the marker and fluorochrome used (e.g. CD4-FITC).
- [x] The axis scales are clearly visible. Include numbers along axes only for bottom left plot of group (a 'group' is an analysis of identical markers).
- [x] All plots are contour plots with outliers or pseudocolor plots.
- [x] A numerical value for number of cells or percentage (with statistics) is provided.

Methodology

Sample preparation	Naive BCC tumors from Mohs surgery patients were minced, then dissociated in 0.5% collagenase in HBSS for up to 1 hour, and 0.05% trypsin for up to 15 minutes. Cells were strained through a 70 um filter, then washed twice using FACS buffer (2% BSA/PBS), then stained with the following antibodies for 30 minutes at 4deg C at 1:100 dilution: anti-LYPD3 (Sino Biological 11836-R213-P), anti-Trop2/TACSTD2 (R&D Systems FAB650-V), anti-LY6d (E48aa1, courtesy of Dr. Ruud Brakenhoff), and anti-CD49f/ItgA6 (Millipore MAB1378). Propidium iodide was used at a concentration of 1:1000 as a dead cell marker.
Instrument	Cells were sorted on a FACSAria II instrument.
Software	BD FACSDiva software was used for data collection. Data was analyzed using FlowJo v10.6.
Cell population abundance	SM+ sorted cells comprised approximately 20-40% of live cells, and SM- sorted cells comprised approximately 40-60% of live cells.
Gating strategy	Abnormally small cells/fragments were first excluded by FSC-A/SSC-A gating, then singlets were isolated by SSC-W/SSC-H and FSC-W/FSC-H gating, then live cells were isolated by negative propidium iodine staining. Live cells were first gated based on positive ItgA6 staining, then Lypd3, Ly6d, and Trop2 staining consecutively. Boundaries between positive and negative staining cell populations were drawn based on distribution of unstained control samples.

- [x] Tick this box to confirm that a figure exemplifying the gating strategy is provided in the Supplementary Information.
